# Supplementary material for: A Likelihood Approach for Real-Time Calibration of Stochastic Compartmental Epidemic Models
Source: PLoS Comput Biol. 2017 Jan 17;13(1):e1005257. doi: 10.1371/journal.pcbi.1005257 (PMC5240920; doi:10.1371/journal.pcbi.1005257)
Supplement: S1 File — (TAR.GZ) [file pcbi.1005257.s014.tar.gz › HSPH_Online-SI-Revision/IPoi09/n100-extreme/n100-extreme_BENCH2_table-4.pdf]

1	newly 3 weeks spec	10 683.	10 379.2	{6349., 12 798.}	20.8635	0.131155	newly 3 weeks cumul	25 079.	24 984.6	{11 427., 35 614.}	31.7506	0.188552	newly 5 weeks spec	10 855.	10 314.6	{7412., 12 637.}	16.699	0.0831993	newly 5 weeks cumul	47 373.	46 248.2	{33 757., 54 736.}	16.6048	0.0963073	newly 58 weeks cumul	85 292.	82 022.4	{74 415., 88 385.}	5.78512	0.0266196
2	newly 3 weeks spec	8383.	7313.63	{5168., 9885.}	21.2217	0.110567	newly 3 weeks cumul	21 608.	24 310.1	{19 912., 29 110.}	20.5969	0.0925245	newly 5 weeks spec	8027.	5162.03	{3229., 8281.}	39.0111	0.234923	newly 5 weeks cumul	38 163.	35 715.2	{27 041., 45 394.}	16.693	0.0815723	newly 54 weeks cumul	72 415.	53 433.5	{37 291., 76 812.}	29.4595	0.162405
3	newly 3 weeks spec	12 915.	12 124.7	{10 482., 13 861.}	11.816	0.074789	newly 3 weeks cumul	29 820.	33 088.	{22 613., 39 188.}	26.9791	0.140101	newly 5 weeks spec	11 421.	9297.26	{6474., 12 286.}	22.825	0.120059	newly 5 weeks cumul	54 424.	53 522.	{49 696., 58 573.}	9.22965	0.0517579	newly 59 weeks cumul	84 212.	79 340.8	{68 510., 89 212.}	8.52465	0.0396382
4	newly 3 weeks spec	15 590.	13 981.	{6940., 19 164.}	27.4793	0.153662	newly 3 weeks cumul	27 674.	26 422.6	{9938., 43 024.}	41.5282	0.219607	newly 5 weeks spec	15 345.	15 505.6	{11 001., 18 909.}	17.8182	0.0799456	newly 5 weeks cumul	61 225.	58 650.5	{43 540., 70 672.}	15.3439	0.0739892	newly 63 weeks cumul	88 766.	90 701.6	{88 195., 93 053.}	3.40705	0.0146897
5	newly 3 weeks spec	10 521.	11 685.6	{8266., 13 870.}	28.1789	0.152945	newly 3 weeks cumul	21 016.	28 168.9	{15 118., 39 149.}	52.7274	0.23368	newly 5 weeks spec	12 782.	10 742.	{7063., 13 763.}	19.2425	0.104918	newly 5 weeks cumul	46 785.	50 807.5	{41 663., 58 637.}	22.1529	0.112164	newly 60 weeks cumul	85 329.	84 438.8	{76 631., 90 882.}	6.05537	0.0277745
6	newly 3 weeks spec	10 702.	10 442.	{8375., 12 372.}	15.7464	0.0990375	newly 3 weeks cumul	23 963.	29 926.3	{16 261., 35 132.}	40.4015	0.191623	newly 5 weeks spec	11 271.	8091.14	{5210., 11 822.}	29.8109	0.169344	newly 5 weeks cumul	47 247.	47 531.2	{39 451., 53 486.}	13.292	0.0765285	newly 58 weeks cumul	86 171.	73 775.5	{59 239., 87 357.}	15.1665	0.0749697
7	newly 3 weeks spec	11 555.	7969.1	{2801., 10 633.}	31.0333	0.235303	newly 3 weeks cumul	26 529.	18 634.7	{5068., 28 024.}	32.4427	0.260702	newly 5 weeks spec	11 237.	9302.92	{7073., 10 704.}	17.2117	0.107606	newly 5 weeks cumul	49 918.	36 963.5	{16 745., 47 463.}	25.9516	0.183523	newly 56 weeks cumul	84 780.	87 237.4	{81 275., 92 311.}	4.94686	0.0210638
8	newly 3 weeks spec	11 018.	10 369.5	{5119., 12 353.}	17.5365	0.132364	newly 3 weeks cumul	24 447.	26 411.	{9187., 35 235.}	35.0037	0.20847	newly 5 weeks spec	11 207.	9513.71	{7039., 12 090.}	18.5118	0.107106	newly 5 weeks cumul	47 781.	46 366.7	{28 664., 53 362.}	16.212	0.107598	newly 58 weeks cumul	87 124.	80 702.2	{70 659., 88 685.}	8.22874	0.0384419
9	newly 3 weeks spec	10 870.	6982.46	{1848., 10 713.}	36.5123	0.298091	newly 3 weeks cumul	22 125.	14 852.8	{3277., 24 936.}	40.6489	0.331612	newly 5 weeks spec	12 694.	9780.44	{6043., 11 460.}	22.9522	0.149574	newly 5 weeks cumul	47 679.	33 432.5	{12 853., 47 422.}	30.7742	0.228086	newly 58 weeks cumul	88 989.	90 496.2	{87 497., 93 570.}	3.09196	0.0133226
10	newly 3 weeks spec	15 442.	13 165.9	{3786., 16 790.}	20.9351	0.158367	newly 3 weeks cumul	29 673.	29 088.7	{5441., 44 026.}	36.4569	0.233616	newly 5 weeks spec	11 765.	11 162.	{7015., 14 744.}	21.9799	0.10549	newly 5 weeks cumul	57 244.	53 462.	{28 357., 62 411.}	15.114	0.0882294	newly 62 weeks cumul	72 720.	75 599.9	{69 879., 79 201.}	5.67166	0.0240573
11	newly 3 weeks spec	9197.	9790.98	{7525., 11 341.}	20.6665	0.106501	newly 3 weeks cumul	23 145.	27 349.	{16 763., 32 553.}	34.4832	0.165219	newly 5 weeks spec	8893.	8300.16	{5697., 10 724.}	18.5071	0.0886408	newly 5 weeks cumul	41 559.	44 942.9	{38 248., 50 590.}	18.9831	0.0909256	newly 56 weeks cumul	75 767.	76 477.7	{61 709., 89 074.}	10.792	0.0475272
12	newly 3 weeks spec	11 654.	6449.18	{1675., 10 321.}	44.6613	0.371796	newly 3 weeks cumul	24 296.	13 899.7	{2984., 24 597.}	44.4083	0.386817	newly 5 weeks spec	12 745.	9043.27	{4946., 10 831.}	29.0445	0.197893	newly 5 weeks cumul	50 388.	31 014.9	{10 932., 45 522.}	38.4479	0.295382	newly 57 weeks cumul	88 506.	88 568.2	{84 319., 91 590.}	2.44895	0.010695
13	newly 3 weeks spec	8371.	9027.08	{7367., 10 288.}	18.1338	0.0917566	newly 3 weeks cumul	19 985.	26 016.8	{18 600., 29 763.}	40.2827	0.171559	newly 5 weeks spec	8948.	7459.74	{5321., 9695.}	20.6659	0.110297	newly 5 weeks cumul	38 014.	41 917.	{36 335., 46 217.}	18.136	0.0862381	newly 55 weeks cumul	83 218.	70 809.7	{57 133., 84 675.}	15.9593	0.0796835
14	newly 3 weeks spec	11 434.	3521.28	{873., 6858.}	69.2034	0.651075	newly 3 weeks cumul	18 644.	6513.59	{1434., 13 072.}	65.0634	0.612135	newly 5 weeks spec	15 310.	7729.31	{3413., 11 546.}	49.5146	0.357794	newly 5 weeks cumul	50 732.	19 806.	{6722., 34 281.}	60.9596	0.504039	newly 60 weeks cumul	70 240.	92 031.4	{90 757., 93 554.}	31.0242	0.117317
15	newly 3 weeks spec	13 412.	10 827.6	{4652., 14 319.}	22.8056	0.169408	newly 3 weeks cumul	27 516.	22 777.6	{7486., 34 675.}	31.5385	0.22153	newly 5 weeks spec	12 056.	12 329.4	{10 259., 14 429.}	14.7652	0.0714319	newly 5 weeks cumul	54 113.	47 650.7	{28 888., 58 977.}	17.1725	0.110252	newly 60 weeks cumul	75 951.	88 046.4	{84 480., 91 131.}	15.9253	0.0639753
16	newly 3 weeks spec	15 726.	10 270.8	{2619., 13 307.}	34.6889	0.252151	newly 3 weeks cumul	30 526.	23 283.6	{4233., 36 805.}	32.3643	0.247069	newly 5 weeks spec	14 011.	11 119.9	{8336., 13 211.}	20.6348	0.113827	newly 5 weeks cumul	61 514.	45 793.3	{18 860., 56 928.}	25.5563	0.166709	newly 60 weeks cumul	88 752.	88 484.8	{81 081., 93 280.}	3.82292	0.0168717
17	newly 3 weeks spec	9053.	7912.32	{3998., 10 332.}	22.9469	0.162158	newly 3 weeks cumul	21 971.	19 369.5	{7802., 29 207.}	32.6675	0.218921	newly 5 weeks spec	9419.	8877.35	{7176., 10 338.}	13.0762	0.0774979	newly 5 weeks cumul	40 887.	36 992.4	{22 535., 46 496.}	20.1627	0.131771	newly 55 weeks cumul	79 924.	84 277.2	{75 270., 91 024.}	8.28801	0.0347774
18	newly 3 weeks spec	8959.	6629.47	{1761., 10 159.}	32.5182	0.258123	newly 3 weeks cumul	19 558.	15 024.2	{3170., 26 110.}	39.5528	0.303749	newly 5 weeks spec	10 392.	8661.1	{5093., 10 326.}	16.68	0.119289	newly 5 weeks cumul	40 376.	31 615.	{11 300., 45 918.}	27.9125	0.206803	newly 56 weeks cumul	75 084.	87 212.	{81 964., 91 279.}	16.1525	0.0645802
19	newly 3 weeks spec	11 412.	6891.04	{1563., 11 224.}	39.6643	0.320132	newly 3 weeks cumul	23 492.	14 829.8	{2693., 27 283.}	43.6678	0.358232	newly 5 weeks spec	12 862.	9639.44	{5443., 11 479.}	25.0549	0.154563	newly 5 weeks cumul	49 674.	33 068.3	{11 074., 49 054.}	33.6048	0.243988	newly 58 weeks cumul	89 565.	90 154.1	{85 430., 93 638.}	3.08118	0.0133934
20	newly 3 weeks spec	15 824.	2910.81	{750., 6388.}	81.6051	0.874433	newly 3 weeks cumul	21 990.	4693.14	{1075., 10 747.}	78.6579	0.818993	newly 5 weeks spec	20 039.	9316.12	{4460., 14 500.}	53.51	0.384742	newly 5 weeks cumul	66 304.	19 771.1	{7373., 36 518.}	70.1812	0.607424	newly 63 weeks cumul	91 232.	91 027.	{86 768., 93 525.}	2.22899	0.00985847
21	newly 3 weeks spec	11 565.	10 987.7	{5265., 14 050.}	24.7861	0.144826	newly 3 weeks cumul	23 774.	24 226.6	{8823., 39 075.}	38.8981	0.208282	newly 5 weeks spec	12 361.	11 912.	{7965., 14 327.}	14.7027	0.0722392	newly 5 weeks cumul	49 979.	48 488.6	{31 268., 58 767.}	17.8017	0.095225	newly 60 weeks cumul	80 337.	84 443.1	{78 115., 90 173.}	7.244	0.0304707
22	newly 3 weeks spec	13 043.	11 280.	{4161., 15 730.}	28.0719	0.203661	newly 3 weeks cumul	25 673.	23 696.	{6372., 40 214.}	42.4483	0.2747	newly 5 weeks spec	13 126.	12 766.2	{8305., 15 712.}	19.4169	0.103418	newly 5 weeks cumul	53 143.	49 556.5	{28 494., 63 355.}	20.476	0.130513	newly 61 weeks cumul	81 637.	87 618.5	{81 540., 91 590.}	7.96565	0.0331041
23	newly 3 weeks spec	8971.	8686.87	{7118., 9837.}	12.2983	0.0812286	newly 3 weeks cumul	22 691.	23 862.6	{15 522., 28 504.}	23.7539	0.13415	newly 5 weeks spec	8717.	7996.52	{6247., 9712.}	14.8026	0.0775569	newly 5 weeks cumul	40 575.	40 453.2	{34 335., 45 140.}	12.2765	0.0731275	newly 54 weeks cumul	74 886.	78 041.9	{66 187., 88 736.}	10.467	0.0442538
24	newly 3 weeks spec	9753.	7530.21	{3757., 9354.}	22.7908	0.158711	newly 3 weeks cumul	22 644.	19 828.8	{7647., 27 305.}	27.6096	0.187336	newly 5 weeks spec	10 377.	7806.05	{5775., 9342.}	24.7755	0.140458	newly 5 weeks cumul	43 672.	35 529.	{20 641., 43 140.}	18.67	0.124966	newly 54 weeks cumul	86 096.	77 604.9	{64 437., 88 574.}	10.8263	0.0522993
25	newly 3 weeks spec	14 475.	4978.13	{927., 8999.}	65.6088	0.603803	newly 3 weeks cumul	28 620.	10 416.9	{1668., 20 176.}	63.6028	0.602235	newly 5 weeks spec	13 481.	7926.16	{3037., 10 336.}	41.2049	0.297206	newly 5 weeks cumul	57 956.	24 955.9	{6466., 41 012.}	56.9399	0.471311	newly 57 weeks cumul	86 463.	89 339.6	{86 436., 91 538.}	3.83104	0.0162987
26	newly 3 weeks spec	8715.	8979.16	{7028., 10 228.}	14.0623	0.0729204	newly 3 weeks cumul	21 621.	24 590.8	{14 325., 30 043.}	29.5714	0.135911	newly 5 weeks spec	8698.	8253.26	{5900., 10 224.}	17.8994	0.0853299	newly 5 weeks cumul	39 423.	41 731.9	{33 656., 46 834.}	14.3399	0.0686217	newly 55 weeks cumul	81 661.	77 677.6	{62 748., 89 543.}	11.18	0.0519049
27	newly 3 weeks spec	9364.	9075.6	{6449., 11 473.}	16.5307	0.0792128	newly 3 weeks cumul	24 919.	29 605.6	{21 126., 33 777.}	26.8854	0.112791	newly 5 weeks spec	8776.	6215.42	{3661., 8596.}	32.8793	0.187069	newly 5 weeks cumul	43 318.	43 471.3	{33 489., 51 389.}	13.4749	0.0616763	newly 56 weeks cumul	79 621.	62 407.8	{43 483., 77 088.}	23.4594	0.12385
28	newly 3 weeks spec	11 755.	9479.77	{4828., 13 375.}	26.6952	0.154311	newly 3 weeks cumul	27 332.	33 705.	{23 712., 39 819.}	31.1864	0.127247	newly 5 weeks spec	11 093.	4277.69	{1480., 7208.}	62.5486	0.496384	newly 5 weeks cumul	50 746.	44 616.	{28 903., 54 962.}	16.5021	0.0872884	newly 60 weeks cumul	87 654.	50 827.2	{31 105., 66 168.}	42.0138	0.251991
29	newly 3 weeks spec	8104.	7621.15	{3416., 9725.}	22.6808	0.159423	newly 3 weeks cumul	20 099.	18 784.7	{6772., 26 363.}	30.3107	0.204601	newly 5 weeks spec	8778.	8434.57	{6909., 9696.}	12.2651	0.0804333	newly 5 weeks cumul	37 699.	35 529.4	{19 254., 43 880.}	19.6886	0.133316	newly 54 weeks cumul	75 236.	83 991.9	{76 810., 90 585.}	12.7303	0.0516566
30	newly 3 weeks spec	13 462.	14 324.7	{9800., 18 254.}	22.3718	0.101112	newly 3 weeks cumul	25 138.	41 564.2	{27 776., 49 296.}	69.4094	0.229313	newly 5 weeks spec	14 527.	6913.34	{2898., 14 201.}	54.5879	0.418642	newly 5 weeks cumul	55 394.	59 270.8	{52 940., 66 712.}	12.7196	0.054268	newly 63 weeks cumul	91 285.	67 377.3	{56 769., 78 578.}	26.1902	0.138107
31	newly 3 weeks spec	13 467.	16 411.2	{7179., 20 968.}	39.2631	0.169574	newly 3 weeks cumul	21 982.	30 983.5	{10 011., 45 669.}	61.5849	0.234425	newly 5 weeks spec	17 282.	13 638.6	{7185., 19 732.}	28.2435	0.158861	newly 5 weeks cumul	58 272.	61 957.7	{48 062., 71 290.}	14.747	0.0650388	newly 64 weeks cumul	87 865.	80 601.3	{72 422., 90 323.}	9.62478	0.0451874
32	newly 3 weeks spec	11 691.	12 578.2	{6990., 14 961.}	24.254	0.126483	newly 3 weeks cumul	23 586.	30 692.4	{11 640., 40 021.}	49.2539	0.214571	newly 5 weeks spec	12 032.	9199.6	{5185., 14 187.}	30.7037	0.171676	newly 5 weeks cumul	48 919.	51 531.9	{38 647., 57 108.}	15.9395	0.0788899	newly 61 weeks cumul	74 173.	68 076.9	{62 724., 73 257.}	8.24124	0.0385124
33	newly 3 weeks spec	9366.	8750.88	{5742., 11 986.}	23.7633	0.120328	newly 3 weeks cumul	20 595.	29 502.8	{20 449., 34 494.}	50.4712	0.189417	newly 5 weeks spec	10 722.	4867.45	{2279., 8215.}	55.9728	0.410063	newly 5 weeks cumul	42 102.	41 147.5	{31 375., 50 198.}	16.6124	0.0786834	newly 59 weeks cumul	87 634.	50 818.4	{36 051., 66 946.}	42.1943	0.252349
34	newly 3 weeks spec	6522.	8311.54	{6294., 9609.}	31.3318	0.120171	newly 3 weeks cumul	16 514.	24 276.2	{18 598., 27 784.}	51.7085	0.185617	newly 5 weeks spec	7135.	6722.84	{4019., 9440.}	25.3343	0.122382	newly 5 weeks cumul	30 872.	38 713.5	{31 808., 43 191.}	28.0146	0.108446	newly 54 weeks cumul	67 088.	60 815.4	{44 570., 74 720.}	15.4793	0.0761135
35	newly 3 weeks spec	10 120.	10 319.6	{6574., 12 604.}	22.0064	0.135024	newly 3 weeks cumul	20 852.	24 307.5	{11 913., 34 867.}	39.0075	0.197368	newly 5 weeks spec	12 158.	10 589.3	{8035., 12 476.}	14.3791	0.0811306	newly 5 weeks cumul	45 344.	45 984.2	{33 710., 54 249.}	17.6902	0.10156	newly 59 weeks cumul	90 553.	86 078.6	{75 761., 91 640.}	5.42622	0.024982
36	newly 3 weeks spec	8518.	6273.	{3703., 10 574.}	35.4232	0.201145	newly 3 weeks cumul	20 967.	24 142.7	{16 495., 34 036.}	26.8247	0.10583	newly 5 weeks spec	8858.	3252.06	{1570., 5675.}	64.7622	0.504316	newly 5 weeks cumul	39 044.	32 008.2	{20 732., 48 297.}	27.9098	0.147192	newly 57 weeks cumul	75 101.	38 474.9	{23 847., 59 625.}	48.9299	0.320054
37	newly 3 weeks spec	14 436.	13 871.6	{7869., 17 265.}	20.6806	0.140542	newly 3 weeks cumul	27 190.	28 029.2	{12 248., 43 681.}	34.1485	0.200277	newly 5 weeks spec	14 344.	13 636.3	{8979., 17 285.}	17.3655	0.0908141	newly 5 weeks cumul	57 920.	57 091.7	{43 773., 67 116.}	14.3261	0.0878622	newly 62 weeks cumul	85 971.	88 006.3	{83 276., 91 073.}	4.01211	0.0172164
38	newly 3 weeks spec	14 671.	6041.14	{1704., 10 952.}	58.8226	0.484047	newly 3 weeks cumul	23 153.	10 635.9	{2625., 20 687.}	55.7093	0.462116	newly 5 weeks spec	16 782.	12 320.8	{7757., 14 653.}	26.583	0.151275	newly 5 weeks cumul	59 731.	32 510.2	{14 158., 49 198.}	45.5722	0.311	newly 62 weeks cumul	80 717.	88 909.9	{82 172., 92 942.}	10.2147	0.0417971
39	newly 3 weeks spec	8107.	8253.66	{3519., 10 687.}	29.0042	0.171712	newly 3 weeks cumul	18 981.	19 547.6	{6682., 28 475.}	36.3862	0.212227	newly 5 weeks spec	8590.	9315.13	{8063., 10 588.}	18.5354	0.0914234	newly 5 weeks cumul	36 334.	38 047.2	{20 537., 47 631.}	26.0898	0.142235	newly 56 weeks cumul	64 192.	84 492.6	{77 061., 89 876.}	31.6249	0.118607
40	newly 3 weeks spec	9375.	9127.84	{5196., 11 946.}	22.1239	0.105846	newly 3 weeks cumul	23 924.	30 608.3	{22 437., 34 809.}	31.1181	0.116696	newly 5 weeks spec	8935.	5062.36	{2109., 7598.}	45.4487	0.300455	newly 5 weeks cumul	42 535.	42 722.5	{27 926., 51 250.}	16.3664	0.0751873	newly 57 weeks cumul	74 746.	52 372.7	{31 566., 66 583.}	29.9622	0.169464
41	newly 3 weeks spec	9002.	11 034.6	{8252., 12 835.}	29.4344	0.117598	newly 3 weeks cumul	21 615.	29 544.1	{18 262., 36 046.}	46.715	0.177052	newly 5 weeks spec	8585.	8630.56	{4783., 12 329.}	26.349	0.121454	newly 5 weeks cumul	39 635.	48 505.3	{41 386., 53 567.}	26.1816	0.103026	newly 58 weeks cumul	64 657.	68 507.1	{54 238., 77 915.}	12.8091	0.0542329
42	newly 3 weeks spec	13 145.	15 045.1	{10 933., 17 638.}	21.8205	0.0885082	newly 3 weeks cumul	25 907.	38 868.1	{21 513., 47 366.}	57.8149	0.199249	newly 5 weeks spec	11 817.	9837.32	{4840., 16 034.}	34.0232	0.185555	newly 5 weeks cumul	52 038.	61 927.5	{51 848., 67 363.}	20.3716	0.0798668	newly 62 weeks cumul	70 332.	77 671.2	{65 890., 88 790.}	13.8421	0.0563777
43	newly 3 weeks spec	10 255.	6347.5	{2143., 9073.}	38.1034	0.298942	newly 3 weeks cumul	22 579.	14 499.3	{4041., 22 191.}	38.7136	0.314987	newly 5 weeks spec	11 209.	8310.12	{5268., 9650.}	25.8621	0.173649	newly 5 weeks cumul	45 260.	30 430.6	{12 723., 41 027.}	32.7649	0.244317	newly 55 weeks cumul	87 509.	87 327.6	{82 759., 91 268.}	3.40371	0.0149638
44	newly 3 weeks spec	15 502.	13 790.5	{10 597., 16 044.}	12.675	0.0770704	newly 3 weeks cumul	33 206.	35 748.2	{18 074., 44 675.}	29.5164	0.150257	newly 5 weeks spec	12 451.	10 617.8	{7198., 15 467.}	24.483	0.124961	newly 5 weeks cumul	61 309.	59 205.5	{49 225., 64 984.}	8.56378	0.0451433	newly 61 weeks cumul	87 960.	84 601.5	{75 753., 92 858.}	6.7042	0.0306112
45	newly 3 weeks spec	16 177.	7953.35	{2264., 13 073.}	50.8354	0.391819	newly 3 weeks cumul	31 598.	15 973.	{3695., 30 053.}	50.1689	0.411794	newly 5 weeks spec	13 010.	11 691.8	{8391., 13 335.}	11.474	0.0711306	newly 5 weeks cumul	61 499.	38 057.3	{16 872., 54 843.}	38.1172	0.256177	newly 60 weeks cumul	80 329.	91 390.5	{87 964., 93 607.}	13.7702	0.0558902
46	newly 3 weeks spec	10 769.	7187.95	{1374., 12 818.}	41.2703	0.338123	newly 3 weeks cumul	20 202.	14 385.2	{2234., 28 694.}	48.2684	0.380902	newly 5 weeks spec	14 108.	10 942.4	{5786., 13 297.}	22.438	0.148246	newly 5 weeks cumul	48 516.	34 881.7	{11 008., 53 649.}	32.7434	0.241773	newly 60 weeks cumul	82 843.	90 399.8	{86 524., 93 112.}	9.20991	0.0380826
47	newly 3 weeks spec	12 425.	9774.06	{5291., 12 859.}	23.8885	0.158073	newly 3 weeks cumul	24 055.	26 286.4	{8940., 34 316.}	34.3497	0.187042	newly 5 weeks spec	13 477.	6053.67	{1680., 12 054.}	55.0815	0.438215	newly 5 weeks cumul	52 391.	40 609.8	{31 743., 46 603.}	22.4871	0.12589	newly 62 weeks cumul	79 732.	49 665.1	{38 169., 56 440.}	37.71	0.209944
48	newly 3 weeks spec	12 536.	8432.88	{1589., 14 405.}	39.6238	0.32905	newly 3 weeks cumul	21 158.	15 254.	{2420., 28 916.}	46.0437	0.36465	newly 5 weeks spec	16 548.	13 474.8	{8551., 15 855.}	18.5715	0.1251	newly 5 weeks cumul	54 873.	40 663.9	{15 132., 58 807.}	28.7635	0.212299	newly 62 weeks cumul	90 499.	88 600.1	{85 105., 92 073.}	2.7388	0.0121192
49	newly 3 weeks spec	21 183.	15 558.8	{7078., 20 893.}	26.6831	0.179698	newly 3 weeks cumul	34 718.	26 796.1	{9578., 41 967.}	33.2594	0.220294	newly 5 weeks spec	14 957.	16 584.6	{11 620., 20 730.}	21.1173	0.0864245	newly 5 weeks cumul	71 735.	62 471.7	{47 380., 72 795.}	13.7004	0.0747265	newly 64 weeks cumul	87 984.	89 568.5	{86 831., 91 993.}	2.4182	0.0103467
50	newly 3 weeks spec	10 269.	8760.82	{5205., 10 552.}	16.1364	0.114662	newly 3 weeks cumul	26 490.	22 165.9	{10 187., 30 450.}	24.6732	0.170729	newly 5 weeks spec	9271.	8995.06	{6863., 10 548.}	14.184	0.0713619	newly 5 weeks cumul	46 170.	40 383.7	{27 697., 47 483.}	14.1322	0.0927364	newly 55 weeks cumul	81 778.	82 806.7	{71 328., 90 805.}	7.36102	0.0321938
